# Cognitive Resources Necessary for Motor Control in Older Adults Are Reduced by Walking and Coordination Training

**DOI:** 10.3389/fnhum.2017.00156

**Published:** 2017-04-11

**Authors:** Ben Godde, Claudia Voelcker-Rehage

**Affiliations:** ^1^Department of Psychology and Methods, Jacobs University BremenBremen, Germany; ^2^Jacobs Center on Lifelong Learning and Institutional Development, Jacobs University BremenBremen, Germany; ^3^Center for Cognitive Science, Bremen UniversityBremen, Germany; ^4^Institute of Human Movement Science and Health, Technische Universität ChemnitzChemnitz, Germany

**Keywords:** motor imagery, functional MRI, motor status, cognitive aging, physical fitness, locomotion

## Abstract

We examined if physical exercise interventions were effective to reduce cognitive brain resources recruited while performing motor control tasks in older adults. Forty-three older adults (63–79 years of age) participated in either a walking (*n* = 17) or a motor coordination (*n* = 15) intervention (1 year, 3 times per week) or were assigned to a control group (*n* = 11) doing relaxation and stretching exercises. Pre and post the intervention period, we applied functional MRI to assess brain activation during imagery of forward and backward walking and during counting backwards from 100 as control task. In both experimental groups, activation in the right dorsolateral prefrontal cortex (DLPFC) during imagery of forward walking decreased from pre- to post-test (Effect size: −1.55 and −1.16 for coordination and walking training, respectively; Cohen’s *d*). Regression analysis revealed a significant positive association between initial motor status and activation change in the right DLPFC (*R*^2^ = 0.243, *F*_(3,39)_ = 4.18, *p* = 0.012). Participants with lowest motor status at pretest profited most from the interventions. Data suggest that physical training in older adults is effective to free up cognitive resources otherwise needed for the control of locomotion. Training benefits may become particularly apparent in so-called dual-task situations where subjects must perform motor and cognitive tasks concurrently.

## Introduction

It has been demonstrated that gait and balance are increasingly in need of cognitive control and supervision with advancing age (Hausdorff et al., 2005; Yogev-Seligmann et al., 2008; Berchicci et al., 2014; Van Swearingen and Studenski, 2014). Such need for a certain amount of cognitive resources for movement coordination or control in older adults (Loewenstein and Acevedo, 2010) has been indicated by expanded brain networks and increased brain activation while performing a single motor task as compared with young adults (for reviews see Seidler et al., 2010; Papegaaij et al., 2014; Hamacher et al., 2015).

Due to the impossibility of performing larger movements in the MR or PET scanner, in recent years, motor imagery has been established as a method to investigate cortical activations during locomotion (Miyai et al., 2001; Malouin et al., 2003; Jahn et al., 2004; la Fougère et al., 2010; Peterson et al., 2014). Numerous studies confirmed that when imagining a movement similar and identical brain areas are activated as if the movement was actually being performed (Stephan et al., 1995; Jeannerod and Frak, 1999; Lotze et al., 1999; Sahyoun et al., 2004; Solodkin et al., 2004; la Fougère et al., 2010; for review see Lafleur et al., 2002; Allali et al., 2014). The locomotor network, as revealed by motor imagery, includes the supplementary and primary motor areas, right prefrontal cortex the basal ganglia, brainstem, tegmentum and cerebellum (Miyai et al., 2001; Jahn et al., 2004; la Fougère et al., 2010; Allali et al., 2014).

With respect to age differences, Allali et al. (2014) observed an age-related increase in brain activity in the right supplementary motor area (BA6), the right orbitofrontal cortex (BA11), and the left dorsolateral frontal cortex (BA10; Allali et al., 2014). Higher activations in older as compared to young adults were also observed in the middle temporal visual area MT/V5 (Wai et al., 2012; Zwergal et al., 2012) and subcortical regions including putamen and substantia nigra (Allali et al., 2014). The resulting use of frontal cortical resources also leads to lower cognitive and motor performance during dual-task situations in older adults (Kahnemann, 1973; Lindenberger et al., 2000; Huxhold et al., 2008; Malcolm et al., 2015).

The amount of cognitive control required for performing a motor task is not only affected by a person’s age but also by her or his motor fitness status (Godde and Voelcker-Rehage, 2010; Berchicci et al., 2014). Using electroencephalography, Berchicci et al. (2014) revealed that older adults who regularly exercise reveal less reliance on extra cognitive control resources during basic visuo-motor functions. In a previous cross-sectional study, using motor imagery, we investigated with functional MRI brain activation in simple and complex walking tasks (walking forward and backward on a treadmill) and analyzed if the motor status of older adults influenced these activation patterns. Motor high-fit individuals showed more activations and larger BOLD signals in motor-related areas compared to low-fit participants but demonstrated lower activity in the dorsolateral prefrontal cortex (DLPFC). Moreover, parietal activation in high-fit participants remained stable throughout the movement period whereas low-fit participants revealed an early drop in activity in this area accompanied by increasing activity in frontal brain regions (Godde and Voelcker-Rehage, 2010).

Based on these findings, one could assume that interventions targeted to improve motor fitness could be a reasonable approach to free up prefrontal (cognitive) brain resources otherwise used for cognitive control of locomotion in older adults. To confirm this assumption, we examined the effects of 1 year of physical exercise interventions on brain activation in the same simple and complex walking tasks (walking forward and backward on a treadmill) as in our previous cross-sectional study. As we could also show previously that different dimensions of physical fitness (cardiovascular and motor fitness) and different types of physical exercise interventions (cardiovascular and motor coordination training) had different positive effects on brain functioning during performance of cognitive tasks (Voelcker-Rehage et al., 2010, 2011), we were also interested whether such interventions would differ in their effect on cognitive control of imagined walking movements. As in the previous study, we used motor imagery of walking forward and backward to assess brain activation patterns during locomotion with functional MRI.

## Materials and Methods

This study was part of the *Old Age on the Move* intervention study at Jacobs University Bremen (see Voelcker-Rehage et al., 2011) that examined effects of different kinds of physical exercise on cognitive, motor, and emotional functioning. Motor status and brain processing during motor imagery were assessed before the start of the intervention (t1) and after 12 months (t2).

### Participants

In total, for the *Old Age on the Move* study, 91 older adults between 63 and 79 years from the Bremen (Germany) area were recruited through the member registry of a German health insurance company (DAK) or through newspaper articles. All participants took part voluntarily and provided written informed consent to the procedures of the study. They received compensation for their travel expenses at the end of the 1-year study amounting to Euro 100. The study conformed to the Code of Ethics of the World Medical Association (Declaration of Helsinki) and was approved by the ethics committee of the German Psychological Society (DGPs; Voelcker-Rehage_072006).

Participants had medical clearance and were screened for health restrictions before inclusion in the study by means of a telephone interview. They were excluded from study participation if they had a history of cardiovascular diseases, any neurological disorder (e.g., self-report of neurological diseases such as a brain tumor, Parkinson’s disease, stroke), any other motor or cognitive restrictions (e.g., a score of less than 27 in the Mini Mental Status Examination, MMSE, Folstein and Van Petten, 2008), or metal devices in the body. Further, participants were screened for number of falls in the year before study participation (no falls: *n* = 38; one fall: *n* = 4, two falls: *n* = 1). Participants who were absent for more than one test day or more than 25% of the training sessions (calculated independently for each half year of the study) were excluded from data analysis (*n* = 47). One participant had to be excluded due to incomplete brain imaging data. None of the included participants experienced change in health status during the 1 year study interval. To assess the subjective ability to perform the requested imagery tasks, the Movement imagery questionnaire MIQ-R (Hall and Martin, 1997) was applied during debriefing directly after the scanning session. The MIQ-R is a rating scale to assess the capacity to elicit mental images. It asks for the clarity of image (scale from 1 very hard to see/feel to 7 very easy to see/feel) and the intensity in which participants could feel themselves making movements (Hall and Martin, 1997). No further participant had to be excluded because of not answering to or scoring less than 4 on the 7-point vividness scale of the questionnaire.

The final sample consisted of 43 participants between 63 and 79 years of age (28 women and 16 men, mean age = 69.6, SD = 3.8). Detailed demographic information as well as information about cognitive and fitness status of the participants is summarized in Table 1. Participants of the experimental and the control groups did not differ statistically on measures of age, years of formal education, intelligence index, health, physical activity index, BMI, hypertension, estrogen replacement therapy (for women only) and positive affect (always *p* > 0.10).

**Table 1:** **Demographic information for participants of the two experimental groups (cardiovascular and coordination training) and the control group**.

	Cardiovascular training		Coordination training		Control group	
Characteristic	*M*	*SD*	*M*	*SD*	*M*	*SD*
Age	68.47	3.06	71.33	4.67	69.27	3.29
Education	13.00	2.96	12.06	3.77	12.09	2.34
IQ	51.42	5.77	47.83	5.36	49.93	5.91
Health	1.35	0.93	1.33	1.18	1.36	1.75
Subj. health	3.94	0.90	3.67	0.49	3.27	0.65
Activity index	1223.38	685.29	1028.29	609.55	1610.55	1044.56
BMI	27.44	4.31	25.88	2.60	26.25	3.43
Hypertension	0.12	0.33	0.50	0.52	0.18	0.41
ERT	0.24	0.44	0.33	0.49	0.09	0.31
Positive affect	3.74	0.67	3.68	0.53	3.76	0.54
VO2 peak	1.62	0.35	1.60	0.31	1.76	0.42

*Age (average age in years), Education (years of education), IQ, Health (number of diseases), Activity Index (kcal expended per week by leisure time and physical activities, see Huy et al., 2008), Body Mass Index (BMI), hypertension (proportion of participants who had been diagnosed with hypertensive disorder), estrogene replacement therapy (ERT, proportion of participants who participated in an estrogene replacement therapy), and positive affect (affect questionnaire encompassing high and low arousal (Kessler and Staudinger, 2009). There was no significant group effect for any of the measures*.

As describe in Voelcker-Rehage et al. (2011), only small sample selectivity was found for age (remaining participants were older), health (remaining participants were healthier), and positive affect (remaining participants were more positive). Given the size of the effects, it seems viable to conclude that findings obtained with the post-test sample may be generalized to the pre-test parent sample.

### Interventions

Participants were assigned to two experimental groups and one control group. Not all interventions could be offered at all training facilities and thus randomization of group assignment was restricted by residency of the participants. Training groups were led by an experienced exercise leader, three times a week and 1 h each for 12 months (Voelcker-Rehage et al., 2011). Participants of the cardiovascular training group (*N* = 17, 12 women, 5 men, mean age = 69.3, SD = 3.3) participated in a walking intervention designed to improve cardiorespiratory fitness (aerobic endurance). Training intensity prescriptions were based on HR responses to spiroergometry exercise testing and was aimed to meet a moderate level. Participants of the second intervention group (*N* = 15, 10 women, 5 men, mean age = 71.3, SD = 4.7) received coordination training designed to improve fine and gross-motor body coordination. This program focused on the improvement of complex movements for the whole body such as balance, eye-hand coordination, leg-arm coordination as well as spatial orientation and reaction to moving objects/persons. The active control group (*N* = 11, 6 women, 5 men, mean age = 68.5, SD = 3.1) performed a program of relaxation techniques, stretching and limbering for the whole body especially designed for older adults. This group served as a control group to evaluate the potential effects of being involved in a guided group activity for 12 months as well as controlling for retest effects. For details of the intervention programs, see Voelcker-Rehage et al. (2011).

### Assessment of Motor Status

The motor status of the participants was assessed at t1 and t2 by a heterogeneous motor test battery comprising tests of the five dimensions movement speed, balance, fine coordination, flexibility and strength (Godde and Voelcker-Rehage, 2010): movement speed was assessed by use of the following four tests: hand tapping (Oja and Tuxworth, 1995; cronbachs α = 0.88), feet tapping (Voelcker-Rehage and Wiertz, 2003; cronbachs α = 0.97), 30-s chair stand test (Rikli and Jones, 1999; single trial), and agility test (Adrian, 1981; cronbachs α = 0.95). Balance was assessed by backwards beam walk (Kiphard and Schilling, 1974; cronbachs α = 0.90) and one-leg-stand with eyes open and closed (Ekdahl et al., 1989, cronbachs α = 0.88). Further we assessed fine coordination by use of the Purdue Pegboard test (Tiffin and Asher, 1948; cronbachs α = 0.93), flexibility by the shoulder flexibility test (Rikli and Jones, 1999; cronbachs α = 0.95) and strength by measuring grip force (Igbokwe, 1992; cronbachs α = 0.97). An overall index for the motor status (mean of the *z*-transformed individual performances within the five domains) was calculated using a *z*-transformed sum score of the five fitness dimensions. This index was normally distributed at T1 (Shapiro-Wilk test: *W*_(43)_ = 0.978, *p* = 0.564).

### Movement Imagery

At t1 and t2, participants performed three imagery tasks with eyes closed and in first-person perspective: (i) walking forward with an individual moderate speed (2.5–3.5 km/h); (ii) walking backward in tandem walk (1 km/h); (iii) standing still and relaxed (baseline condition); and (iv) counting backward from 100 was chosen as a non-movement control condition. Outside the MR scanner, before the test sessions, a standardized description of the imagery tasks was provided and participants completed a task familiarization exercise. Participants were trained in the two experimental motor tasks (walking forward and backward) and the two control tasks (standing and counting backward). First, participants performed the real tasks and the imagination on a treadmill (Model Lode Valiant, Groningen Netherland). Walking forward was trained with an individual moderate speed (2.5–3.5 km/h) and easy swinging of the arms and walking backward was trained in tandem walk (1 km/h). All participants were trained as long as they needed to feel comfortable on the treadmill. The range was between 10 min and 20 min in total. We used a treadmill instead of real-world walking to provide constant visual input and ground. After executing the real and imagined movements on the treadmill participants trained imagination of these movements (including gait initiation) in a horizontal position in periods of 20 s each until they felt well experienced with the tasks. Participants were instructed to close their eyes and to use a first-person perspective to perform the imagery tasks. Then, at another day, participants first repeated the movement imagination outside the MRI scanner until they felt confident again and then performed the tasks within the scanner (first person perspective, eyes closed).

### Functional MRI

Functional MRI scans were performed at pre- and post-test in a randomized block design with six blocks of 20 s for each of the four conditions in a randomized order without any break between the blocks resulting in a total of 24 blocks lasting for 480 s.

We used a 3T head scanner (Siemens Magnetom Allegra, Erlangen, Germany). A T2*-weighted gradient echo multislice sequence (EPI, TR 2500 ms, TE 60 ms, voxel size 3 × 3 × 3 mm, matrix 64 × 64) was used to acquire 48 slices covering the whole brain and the cerebellum. Additionally, a high-resolution T1-weighted anatomical 3D-dataset containing 172 sagittal slices (1 × 1 × 1 mm^3^) was acquired for each subject.

Analysis of fMRI data was performed using Brain Voyager (Brain Innovation B.V., Maastricht, Netherlands). FMRI data were first corrected for motion artifacts and linear trends, smoothed in the temporal (2.8 s) and spatial (6 mm) domain, and normalized to Talairach space. The BOLD responses were modeled with a delayed box-car function convolved with a canonical hemodynamic response and a general linear model (GLM) was applied to the time course of each voxel. A random effects analysis was performed, considering the inter-subject variability; the results can therefore be generalized to other samples. On the first level, weighted beta-images were computed for every condition (forward walking, backward walking, and counting backward from 100) relative to baseline (standing still). On the second level, these individual beta values were then entered into a 3 (INTERVENTION groups) × 2 (SESSION: t1 vs. t2) × 3 (CONDITION) random effects analysis of variance *P*-values were corrected for multiple comparisons by false discovery rate (FDR, *P* < 0.05) and cluster threshold estimation using Monte Carlo simulations (alpha level < 0.05; Forman et al., 1995; Goebel et al., 2006). Effect sizes of group differences (intervention groups vs. control group) in cortical activation changes (t2–t1) were calculated as Cohen’s *d* (based on sample size; Hedge’s Adjustment and weighted average).

### Further Statistical Analysis

Statistical analyses were performed using SPSS for Windows version 20 (IBM Corp., Armonk, NY, USA). From those regions revealing a significant INTERVENTION × SESSION × CONDITION interaction effect we selected those which in our previous cross-sectional study also revealed to be related to fitness (Godde and Voelcker-Rehage, 2010). BOLD values and beta estimates of the individual peak voxels in these regions were extracted and subjected to linear regression analysis with following regressors: group (experimental or control, dummy coded as 1 or −1, respectively; because both intervention groups did not differ in their effect on brain activation change in these regions we combined them in this analysis), the interaction term of group and initial motor status at t1, and the interaction term of group and change in motor status from t1 to t2 (Table 2). For that purpose, motor status indices at t1 and t2 were *z*-transformed. T2 values were transformed relative to t1 and change in motor status was defined as the difference t2−t1 of these *z*-transformed indices. For calculating the interaction terms with factor group both indices were centered. The level of significance was set to *p* < 0.05.

**Table 2:** **Effects of interventions on motor fitness (*z*-scores related to t1)**.

	Cardiovascular training		Coordination training		Control group	
	*M*	*SD*	*M*	*SD*	*M*	*SD*
Motor fit t1	0.11	2.63	−0.19	2.49	0.09	1.49
Motor fit t2	1.13	3.06	1.31	2.73	1.33	2.06

## Results

To answer our research questions, fMRI data obtained during motor imagery were analyzed in a two-step procedure. First, we identified regions that revealed significant INTERVENTION × SESSION × CONDITION interaction effects. This interaction effect was revealed for a variety of frontal, parietal, and subcortical brain regions. These regions included frontally the right DLPFC and middle frontal cortex, bilaterally the superior and medial frontal gyrus (MeFG), the precentral gyrus (PrCG) and the left anterior cingulate. Further the postcentral gyrus (PoCG) and the left caudate revealed such interaction effects (Table 3).

**Table 3:** **Regions of interests (ROI) with significant SESSION × INTERVENTION × CONDITION interaction Effects (*P* < 0.05, cluster threshold: 37 voxels)**.

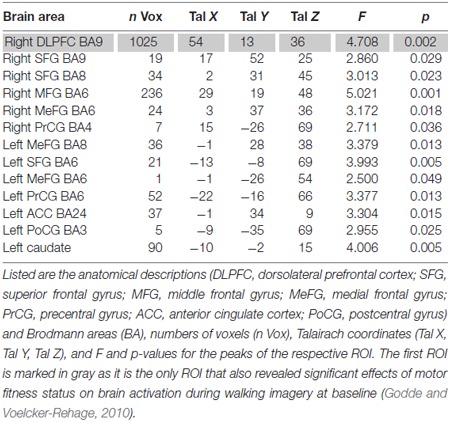

In the second step, from those regions, we selected only regions that had also been activated stronger in less- than in higher motor fit participants in our previous cross-sectional study (Godde and Voelcker-Rehage, 2010), thus indicating increased need for cognitive control of motor imagery in low-fit older adults. Only the right DLPFC (Brodmann area 9) met this second criterion.

**Figure 1:**
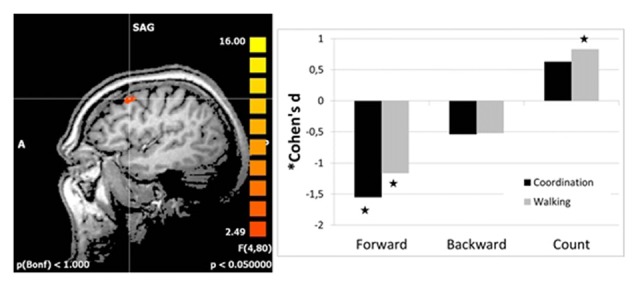
**Effect sizes for group differences in activation change (change in beta estimates) in the right dorsolateral prefrontal cortex (DLPFC; left panel).** Effect sizes of intervention groups (coordination and walking group) relative to the control group were calculated as Cohen’s d (based on sample size; Hedge’s Adjustment and weighted average). Stars indicate significant effects of the intervention groups.

Follow-up analyses revealed significant reductions in right DLPFC activation from t1 to t2 for both intervention groups as compared to the control group for imagery of walking forward. Even activation for backward walking was reduced in both intervention groups, but not significantly. Interestingly, DLPFC activation was increased for the walking group but not for the coordination group as compared to the control group for counting backward from 100 (Figure 1). There were no differences in effect size between the two interventions as revealed by direct comparison of both intervention groups (pairwise two-tailed paired samples *t*-test, *p* > 0.13).

**Figure 2:**
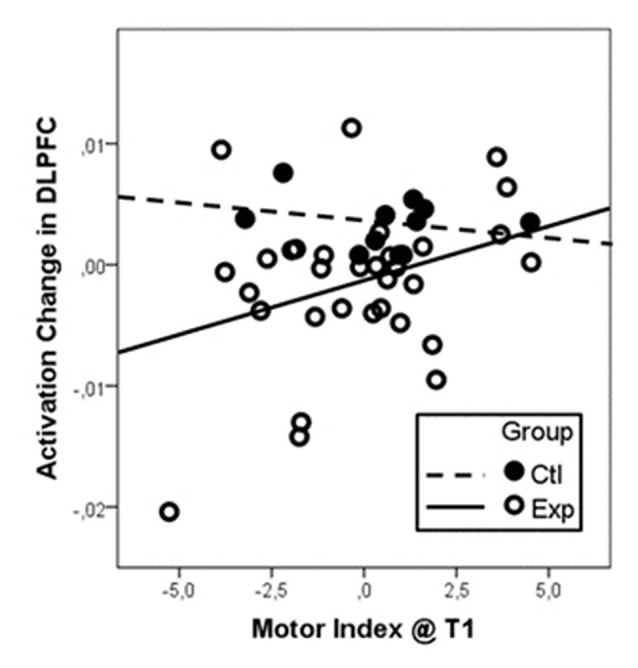
**Activation change in the right DLPFC dependent on baseline motor fitness as indicated as the motor index at t1.** Data were centered and *z*-transformed. Since we did not find differential effects of the two intervention types (walking and coordination), data from both groups were pooled and compared to the control group. Particularly participants with low motor index at t1 (low motor index) revealed the strongest reduction in DLPFC activation after the intervention (negative change values).

Regression analysis with initial motor status at T1 and change in motor status from T1 to T2 as regressors revealed a significant positive association of the initial motor status and activation change in right DLPFC for pooled intervention groups. The overall linear regression model was significant (*R*^2^ = 0.243, *F*_(3,39)_ = 4.18, *p* = 0.012). Besides the factor group (standardized beta coefficient = −0.39, *T* = −2.76, *p* = 0.009), the interaction of group and baseline motor index (standardized beta coefficient = 0.33, *T* = 2.30, *p* = 0.027) were revealed as significant predictors for change in right DLPFC activation. As illustrated in Figure 2, participants with low motor status at t1 profited most from the intervention. Because of the small sample size, however, these results must be taken with care.

## Discussion

Our study addressed the question whether physical training interventions are effective to reduce the need for cognitive control of locomotion in older adults. Results confirm that both walking and coordination training reduced frontal brain activation during imagery of walking forward and backward. Moreover, participants with lower baseline motor status profited most from the intervention. Our data suggest that physical interventions not only have direct effects on cognitive and brain function in older adults as reported earlier (Colcombe and Kramer, 2003; Colcombe et al., 2004; Hillman et al., 2008; Lustig et al., 2009; Voelcker-Rehage et al., 2011; Hayes et al., 2013; Voelcker-Rehage and Niemann, 2013; Schättin et al., 2016), but also indirect effects by freeing up frontal brain resources otherwise needed for the control of motor actions. Herewith our findings are also in line with a recent study using near-infrared spectroscopy revealing that video game dancing training and balance training reduce left and right PFC oxygenation during fast walking (Eggenberger et al., 2016). With decreasing reserve capacity in older adults, these effects may become increasingly important and become especially apparent in so-called dual-task situations where subjects have to perform motor and cognitive tasks concurrently, for example, during crossing a street while observing the traffic flow or walking by talking (Lindenberger et al., 2000; Yogev-Seligmann et al., 2010; Al-Yahya et al., 2011; Neider et al., 2011).

We found INTERVENTION × SESSION × CONDITION interaction effects for a variety of frontal cortical areas belonging to the motor imagery network as described earlier (Allali et al., 2014; Hamacher et al., 2015), indicating altered use of cognitive resources for motor control after the training interventions. Interestingly, these effects were also found for the right but not the left DLPFC which has also been shown to be involved in motor imagery in previous studies (e.g., Malouin et al., 2003; Jahn et al., 2004), particularly in older adults (Allali et al., 2014). These studies, however, did not consider the motor fitness status of the participants. When the motor fitness status was considered, as in our previous cross-sectional study (Godde and Voelcker-Rehage, 2010), more activity in the right DLPFC was revealed in low-fit as compared to high-fit older adults. We explained this finding in the sense that the control condition (standing still) also requires some attentional control and thus the right DLPFC activity particularly seen in low-fit participants only mirrors additional activation that can be interpreted as compensatory. Such additional activity in homologs contralateral frontal areas in older adults has repeatedly been put into the context of compensatory mechanisms of age-related changes (e.g., “Hemispheric asymmetry reduction in older adults (HAROLD)” hypothesis; Cabeza, 2002). However, it is not possible to measure motor performance using a motor imagery paradigm, and therefore it must remain open if this additional frontal activation reflects compensation, dedifferentiation or just the higher task complexity for low as compared to high-fit participants. Increased involvement of prefrontal cortex in older adults during complex gait tasks or in imagined walking conditions with high cognitive load was also confirmed by recent reviews (Holtzer et al., 2014; Hamacher et al., 2015).

Interestingly, the interventions did not differ (but they differed from the control group) in respect to their effects on cognitive resources allocated in the DLPFC for the control of walking movements. One explanation might be that the effect in the walking group similar to the coordination group was due to better motor control abilities rather than enhanced cardiovascular fitness, i.e., due to the extensive walking experience walking became more automated (Ross et al., 2003; Wei and Luo, 2010). This is supported by the finding that effects are stronger for imagery of walking forward, what has specifically been trained in the walking group, than walking backwards, what is the more complex task. Indeed, recent MR studies revealed motor training-induced gray and white matter changes in motor-related areas such as the supplementary and presupplementary motor cortex (SMA/pre-SMA) and increased functional connectivity to prefrontal and parietal brain regions, even in older adults (for review see Taubert et al., 2012). Further, coordinative exercise as applied here leads to increased basal ganglia volume in older adults (Niemann et al., 2014).

It might be that (additionally) increase in cardiovascular fitness also could have led to some positive effects on cognitive processing based on more efficient use of frontal brain resources (Voelcker-Rehage et al., 2011). With the paradigms tested here, however, that does not seem to play a role. This might be different under dual-task conditions but must remain speculative here.

We aimed to assure clarity and intensity of motor imagery during scanning. For that purpose, we applied the MIQ-R only. A chronometric test in which the time needed to complete real walking and walking imagery is compared for the two conditions could have given supportive evidence on better forward walking abilities in the walking group.

The motor fitness status of the control group greatly improved from time 1 to time 2, even more so than the cardiovascular training group (Table 2) and one might wonder why this improvement in actual motor fitness was not reflected by a change in brain activity (specifically in right DLPFC) for imagined movements in the control group as well. It could well be that stretching and relaxation as exercised in the control group might improve proprioceptive function and self-perception. However, the control group did not explicitly train walking or actively controlled movements. This might explain why they generally performed better in the motor test battery but did not reveal activation changes related to specific motor control in frontal brain regions.

The reader might also wonder about positive effect sizes for counting (Figure 1) which seems to indicate increased activation after the intervention and thus more need for cognitive resources. However, all effects were calculated in contrast to the standing condition as baseline condition. Thus, this positive effect for counting might be due to a negative effect for standing (though much less than for walking backwards or even more so walking forward).

Further experiments using electroencephalography or near-infrared spectroscopy during real movements might add additional evidence that frontal brain resources used for cognitive control can be reduced by specific motor training in older adults (Eggenberger et al., 2016; Schättin et al., 2016). Overall physical activity that stress the motor system (here either by regular walking or coordination training) might be beneficial to preserve or enhance cognitive resources (see Voelcker-Rehage et al., 2011), but also to preserve motor functioning—at least in the practiced tasks (here walking forward) leading to less cognitive resources needed to perform a motor task and having resources available in complex situations of daily life.

Based on our results, it is difficult to favor one intervention (walking vs. coordination training) over the other and it might be advised to combine both exercise dimensions in training programs for older adults.

## Author Contributions

BG and CVR designed and performed the study and acquired, analyzed and interpreted the data. They together drafted the manuscript and agreed to be accountable for all aspects of the work in terms of accuracy and integrity.

## Conflict of Interest Statement

The authors declare that the research was conducted in the absence of any commercial or financial relationships that could be construed as a potential conflict of interest.

## References

[B101] Adrian M. J. (1981). “Flexibility in the aging adult,” in Exercises and Aging: The Scientific Basic, eds Smith E. L. Serfass R. C. (Hillside, NJ: Enslow Publishers), 45–58.

[B1] Allali G. van der Meulen M. Beauchet O. Rieger S. W. Vuilleumier P. Assal F. (2014). The neural basis of age-related changes in motor imagery of gait: an fMRI study. J. Gerontol. A Biol. Sci. Med. Sci. 69, 1389–1398. 10.1093/gerona/glt207 24368777

[B2] Al-Yahya E. Dawes H. Smith L. Dennis A. Howells K. Cockburn J. (2011). Cognitive motor interference while walking: a systematic review and meta-analysis. Neurosci. Biobehav. Rev. 35, 715–728. 10.1016/j.neubiorev.2010.08.008 20833198

[B3] Berchicci M. Lucci G. Perri R. L. Spinelli D. Di Russo F. (2014). Benefits of physical exercise on basic visuo-motor functions across age. Front. Aging Neurosci. 6:48. 10.3389/fnagi.2014.00048 24672482 PMC3955899

[B4] Cabeza R. (2002). Hemispheric asymmetry reduction in older adults: the HAROLD model. Psychol. Aging 17, 85–100. 10.1037/0882-7974.17.1.85 11931290

[B5] Colcombe S. Kramer A. F. (2003). Fitness effects on the cognitive function of older adults: a meta-analytic study. Psychol. Sci. 14, 125–130. 10.1111/1467-9280.t01-1-01430 12661673

[B6] Colcombe S. J. Kramer A. F. Erickson K. I. Scalf P. McAuley E. Cohen N. J.. (2004). Cardiovascular fitness, cortical plasticity, and aging. Proc. Natl. Acad. Sci. U S A 101, 3316–3321. 10.1073/pnas.0400266101 14978288 PMC373255

[B7] Eggenberger P. Wolf M. Schumann M. de Bruin E.D. (2016). Exergame and balance training modulate prefrontal brain activity during walking and enhance executive function in older adults. Front. Aging Neurosci. 8:66. 10.3389/fnagi.2016.00066 27148041 PMC4828439

[B102] Ekdahl C. Jarnlo G. B. Andersson S. I. (1989). Standing balance in healthy subjects. Evaluation of a quantitative test battery on a force platform. Scand. J. Rehabil. Med. 21, 187–195. 2631193

[B8] Folstein J. R. Van Petten C. (2008). Influence of cognitive control and mismatch on the N2 component of the ERP: a review. Psychophysiology 45, 152–170. 10.1111/j.1469-8986.2007.00602.x 17850238 PMC2365910

[B9] Forman S. D. Cohen J. D. Fitzgerald M. Eddy W. F. Mintun M. A. Noll D. C. (1995). Improved assessment of significant activation in functional magnetic resonance imaging (fMRI): use of a cluster-size threshold. Magn. Reson. Med. 33, 636–647. 10.1002/mrm.1910330508 7596267

[B10] Godde B. Voelcker-Rehage C. (2010). More automation and less cognitive control of imagined walking movements in high-versus low-fit older adults. Front. Aging Neurosci. 2:139. 10.3389/fnagi.2010.00139 20877433 PMC2944669

[B11] Goebel R. Esposito F. Formisano E. (2006). Analysis of functional image analysis contest (FIAC) data with Brainvoyager QX: from single-subject to cortically aligned group general linear model analysis and self-organizing group independent component analysis. Hum. Brain Mapp. 27, 392–401. 10.1002/hbm.20249 16596654 PMC6871277

[B12] Hall C. R. Martin K. A. (1997). Measuring movement imagery abilities: a revision of the movement imagery questionnaire. J. Ment. Imagery 21, 143–154.

[B13] Hamacher D. Herold F. Wiegel P. Hamacher D. Schega L. (2015). Brain activity during walking: a systematic review. Neurosci. Biobehav. Rev. 57, 310–327. 10.1016/j.neubiorev.2015.08.002 26306029

[B14] Hausdorff J. M. Yogev G. Springer S. Simon E. S. Giladi N. (2005). Walking is more like catching than tapping: gait in the elderly as a complex cognitive task. Exp. Brain Res. 164, 541–548. 10.1007/s00221-005-2280-3 15864565

[B15] Hayes S. M. Hayes J. P. Cadden M. Verfaellie M. (2013). A review of cardiorespiratory fitness-related neuroplasticity in the aging brain. Front. Aging Neurosci. 5:31. 10.3389/fnagi.2013.00031 23874299 PMC3709413

[B16] Hillman C. H. Erickson K. I. Kramer A. F. (2008). Be smart, exercise your heart: exercise effects on brain and cognition. Nat. Rev. Neurosci. 9, 58–65. 10.1038/nrn2298 18094706

[B17] Holtzer R. Epstein N. Mahoney J. R. Izzetoglu M. Blumen H. M. (2014). Neuroimaging of mobility in aging: a targeted review. J. Gerontol. A Biol. Sci. Med. Sci. 69, 1375–1388. 10.1093/gerona/glu052 24739495 PMC4204614

[B18] Huxhold O. Schäfer S. Lindenberger U. (2008). Wechselwirkungen zwischen sensomotorik und kognition im alter. Z. Gerontol. Geriat. 42, 93–98. 10.1007/s00391-008-0566-3 18925357

[B103] Huy C. Becker S. Gomolinsky U. Klein T. Thiel A. (2008). Health, medical risk factors, and bicycle use in everyday life in the over-50 population. J. Aging Phys. Act. 16, 454–464. 10.1123/japa.16.4.454 19033605

[B104] Igbokwe N. U. (1992). Hand grip dynamometer and arm muscle size in teenage boys and girls. J. Phys. Educ. Sport Sci. 4, 15–19.

[B19] Jahn K. Deutschländer A. Stephan T. Strupp M. Wiesmann M. Brandt T. (2004). Brain activation patterns during imagined stance and locomotion in functional magnetic resonance imaging. Neuroimage 22, 1722–1731. 10.1016/j.neuroimage.2004.05.017 15275928

[B105] Jeannerod M. Frak V. (1999). Mental imaging of motor activity in humans. Curr. Opin. Neurobiol. 9, 735–739. 10.1016/s0959-4388(99)00038-0 10607647

[B20] Kahnemann D. (1973). Attention and Effort. Englewood Cliffs, NJ: Prentice Hall.

[B106] Kessler E. M. Staudinger U. M. (2009). Affective experience in adulthood and old age: the role of affective arousal and perceived affect regulation. Psychol. Aging 24, 349–362. 10.1037/a0015352 19485653

[B107] Kiphard E. J. Schilling F. (1974). Körperkoordinationstest Für Kinder [Body Coordination Test for Children]. Weinheim: Beltz. 5511840

[B108] Lafleur M. F. Jackson P. L. Malouin F. Richards C. L. Evans A. C. Doyon J. (2002). Motor learning produces parallel dynamic functional changes during the execution and imagination of sequential foot movements. Neuroimage 16, 142–157. 10.1006/nimg.2001.1048 11969325

[B21] la Fougère C. Zwergal A. Rominger A. Förster S. Fesl G. Dieterich M.. (2010). Real versus imagined locomotion: a [^18^F]-FDG PET-fMRI comparison. Neuroimage 50, 1589–1598. 10.1016/j.neuroimage.2009.12.060 20034578

[B22] Lindenberger U. Marsiske M. Baltes P. B. (2000). Memorizing while walking: increase in dual-task costs from young adulthood to old age. Psychol. Aging 15, 417–436. 10.1037/0882-7974.15.3.417 11014706

[B109] Loewenstein D. Acevedo A. (2010). “The relationship between instrumental activities of daily living and neuropsychological performance,” in Neuropsychology of Everyday Functioning, eds Marcotte T. D. Grant I. (New York, NY: The Guilford Press), 93–112.

[B110] Lotze M. Montoya P. Erb M. Hülsmann E. Flor H. Klose U.. (1999). Activation of cortical and cerebellar motor areas during executed and imagined hand movements: an fMRI study. J. Cogn. Neurosci. 11, 491–501. 10.1162/089892999563553 10511638

[B24] Lustig C. Shah P. Seidler R. Reuter-Lorenz P. A. (2009). Aging, training, and the brain: a review and future directions. Neuropsychol. Rev. 19, 504–522. 10.1007/s11065-009-9119-9 19876740 PMC3005345

[B25] Malcolm B. R. Foxe J. J. Butler J. S. De Sanctis P. (2015). The aging brain shows less flexible reallocation of cognitive resources during dual-task walking: a mobile brain/body imaging (MoBI) study. Neuroimage 117, 230–242. 10.1016/j.neuroimage.2015.05.028 25988225 PMC5080979

[B26] Malouin F. Richards C. L. Jackson P. L. Dumas F. Doyon J. (2003). Brain activations during motor imagery of locomotor-related tasks: a PET study. Hum. Brain Mapp. 19, 47–62. 10.1002/hbm.10103 12731103 PMC6872050

[B27] Miyai I. Tanabe H. C. Sase I. Eda H. Oda I. Konishi I.. (2001). Cortical mapping of gait in humans: a near-infrared spectroscopic topography study. Neuroimage 14, 1186–1192. 10.1006/nimg.2001.0905 11697950

[B28] Neider M. B. Gaspar J. G. McCarley J. S. Crowell J. A. Kaczmarski H. Kramer A. F. (2011). Walking and talking: dual-task effects on street crossing behavior in older adults. Psychol. Aging 26, 260–268. 10.1037/a0021566 21401262 PMC3699858

[B29] Niemann C. Godde B. Staudinger U. Voelcker-Rehage C. (2014). Exercise-induced changes in basal ganglia volume and cognition in older adults. Neuroscience 281, 147–163. 10.1016/j.neuroscience.2014.09.033 25255932

[B111] Oja P. Tuxworth B. (1995). Eurofit for Adults—Assessment of Health-Related Fitness. Tampere: Council of Europe Publishing.

[B30] Papegaaij S. Taube W. Baudry S. Otten E. Hortobágyi T. (2014). Aging causes a reorganization of cortical and spinal control of posture. Front. Aging Neurosci. 6:28. 10.3389/fnagi.2014.00028 24624082 PMC3939445

[B31] Peterson D. S. Pickett K. A. Duncan R. P. Perlmutter J. S. Earhart G. M. (2014). Brain activity during complex imagined gait tasks in Parkinson disease. Clin. Neurophysiol. 125, 995–1005. 10.1016/j.clinph.2013.10.008 24210997 PMC3981914

[B112] Rikli R. E. Jones C. J. (1999). Development and validation of a functional fitness test for community-residing older adults. J. Aging Phys. Act. 7, 129–161. 10.1123/japa.7.2.129

[B113] Ross J. S. Tkach J. Ruggieri P. M. Lieber M. Lapresto E. (2003). The mind’s eye: functional MR imaging evaluation of golf motor imagery. AJNR Am. J. Neuroradiol. 24, 1036–1044. 12812924 PMC8149015

[B114] Sahyoun C. Floyer-Lea A. Johansen-Berg H. Matthews P. M. (2004). Towards an understanding of gait control: brain activation during the anticipation, preparation and execution of foot movements. Neuroimage 21, 568–575. 10.1016/j.neuroimage.2003.09.065 14980558

[B32] Schättin A. Arner R. Gennaro F. de Bruin E. D. (2016). Adaptations of prefrontal brain activity, executive functions, and gait in healthy elderly following exergame and balance training: a randomized controlled study. Front. Aging Neurosci. 8:278. 10.3389/fnagi.2016.00278 27932975 PMC5120107

[B33] Seidler R. D. Bernard J. A. Burutolu T. B. Fling B. W. Gordon M. T. Gwin J. T.. (2010). Motor control and aging: links to age-related brain structural, functional, and biochemical effects. Neurosci. Biobehav. Rev. 34, 721–733. 10.1016/j.neubiorev.2009.10.005 19850077 PMC2838968

[B115] Solodkin A. Hlustik P. Chen E. E. Small S. L. (2004). Fine modulation in network activation during motor execution and motor imagery. Cereb. Cortex 14, 1246–1255. 10.1093/cercor/bhh086 15166100

[B116] Stephan K. M. Fink G. R. Passingham R. E. Silbersweig D. Ceballos-Baumann A. O. Frith C. D.. (1995). Functional anatomy of the mental representation of upper extremity movements in healthy subjects. J. Neurophysiol. 73, 373–386. 7714579 10.1152/jn.1995.73.1.373

[B34] Taubert M. Villringer A. Ragert P. (2012). Learning-related gray and white matter changes in humans an update. Neuroscientist 18, 320–325. 10.1177/1073858411419048 22013150

[B117] Tiffin J. Asher E. J. (1948). The Purdue pegboard; norms and studies of reliability and validity. J. Appl. Psychol. 32, 234–247. 10.1037/h0061266 18867059

[B35] Van Swearingen J. M. Studenski S. A. (2014). Aging, motor skill, and the energy cost of walking: implications for the prevention and treatment of mobility decline in older persons. J. Gerontol. A Biol. Sci. Med. Sci. 69, 1429–1436. 10.1093/gerona/glu153 25182600 PMC4271095

[B36] Voelcker-Rehage C. Godde B. Staudinger U. M. (2011). Cardiovascular and coordination training differentially improve cognitive performance and neural processing in older adults. Front. Hum. Neurosci. 5:26. 10.3389/fnhum.2011.00026 21441997 PMC3062100

[B37] Voelcker-Rehage C. Godde B. Staudinger U. M. (2010). Physical and motor fitness are both related to cognition in old age. Eur. J. Neurosci. 31, 167–176. 10.1111/j.1460-9568.2009.07014.x 20092563

[B38] Voelcker-Rehage C. Niemann C. (2013). Structural and functional brain changes related to different types of physical activity across the life span. Neurosci. Biobehav. Rev. 37, 2268–2295. 10.1016/j.neubiorev.2013.01.028 23399048

[B118] Voelcker-Rehage C. Wiertz O. (2003). Die Lernfaehigkeit Sportmotorischer Fertigkeiten im Lichte der Entwicklungspsychologie der Lebensspanne [Motor Skill Learning in Focus of Lifespan Developmental Psychology]. Bielefeld: Bielefelder Reihe, Universität Bielefeld.

[B39] Wai Y.-Y. Wang J.-J. Weng Y.-H. Lin W.-Y. Ma H.-K. Ng S.-H.. (2012). Cortical involvement in a gait-related imagery task: comparison between Parkinson’s disease and normal aging. Parkinsonism Relat. Disord. 18, 537–542. 10.1016/j.parkreldis.2012.02.004 22436654

[B40] Wei G. Luo J. (2010). Sport expert’s motor imagery: functional imaging of professional motor skills and simple motor skills. Brain Res. 1341, 52–62. 10.1016/j.brainres.2009.08.014 19686705

[B41] Yogev-Seligmann G. Hausdorff J. M. Giladi N. (2008). The role of executive function and attention in gait. Mov. Disord. 23, 329–342; quiz 472. 10.1002/mds.21720 18058946 PMC2535903

[B42] Yogev-Seligmann G. Rotem-Galli Y. Mirelman A. Dickstein R. Giladi N. Hausdorff J. (2010). How does explicit prioritization alter walking during dual-task performance? Effects of age and sex on gait speed and variability. Phys. Ther. 90, 177–186. 10.2522/ptj.20090043 20023000 PMC2816029

[B43] Zwergal A. Linn J. Xiong G. Brandt T. Strupp M. Jahn K. (2012). Aging of human supraspinal locomotor and postural control in fMRI. Neurobiol. Aging 33, 1073–1084. 10.1016/j.neurobiolaging.2010.09.022 21051105

